# Identification of a locus conferring dominant resistance to maize rough dwarf disease in maize

**DOI:** 10.1038/s41598-018-21677-3

**Published:** 2018-02-19

**Authors:** Ronggai Li, Wei Song, Baoqiang Wang, Jianghao Wang, Dongmin Zhang, Quanguo Zhang, Xinghua Li, Jianfen Wei, Zengyu Gao

**Affiliations:** Key Laboratory of Crop Genetics and Breeding of Hebei Province, Institute of Cereal and Oil Crops, Hebei Academy of Agriculture and Forestry Sciences, Shijiazhuang, 050035 China

## Abstract

Maize rough dwarf disease (MRDD) is a severe viral disease of maize that occurs worldwide, particularly in the summer maize-growing areas in China, resulting in yield losses and quality deterioration in susceptible maize varieties. An effective solution to control MRDD is to use resistance genes to improve the behavior of susceptible genotypes. Here, we employed maize F_2_ populations derived from a cross between susceptible line S221 and resistant line K36 for the deep sequencing of the two DNA pools containing extremely resistant and susceptible F_2_ individuals, and used traditional linkage analysis to locate the resistance-related genomic region. The results showed that MRDD resistance in K36 was controlled by a single dominant locus, and an associated region was identified within the genomic interval of 68,396,487 bp and 69,523,478 bp on chromosome 6. Two simple sequence repeat (SSR) markers 6F29R29 and 6F34R34 were tightly linked to the MRDD resistance locus. The findings of the present study improve our understanding of the inheritance patterns of MRDD resistance, and should inform MRDD-resistant maize breeding programs.

## Introduction

Maize rough dwarf disease (MRDD) is a global viral disease in maize (*Zea mays* L.). It is particularly prevalent in the summer maize-growing areas in China and can lead to more than 30% reduction in yield, and can even result in 100% yield loss in severely infected fields^1–3^. This disease was first detected in 1949 in Italy. Four members of the family Reoviridae belonging to the genus *Fijivirus* are the causal pathogens of MRDD, and include maize rough dwarf virus (MRDV), Mal de Rio Cuarto virus (MRCV), rice black streaked dwarf virus (RBSDV), and southern rice black streaked dwarf virus (SRBSDV). However, the virus strains vary between different regions. MRDV affects maize in Europe, MRCV in South America, and RBSDV and SRBSDV in East Asia^4–8^. These closely related viruses are transmitted in a persistent manner by planthoppers (*Laodelphax striatellus*) that infect common plant hosts such as rice, wheat, and maize. In China, RBSDV and SRBSDV are the pathogens of MRDD that can be transmitted from infected wheat to maize by planthoppers^6^. MRDD is particularly severe in the Huang-Huai-Hai River summer maize-growing region when maize is sown around the wheat harvesting period^1^.

MRDD symptoms typically include dwarfed plant height, dark-green leaves that appear rough due to waxy enations, malformed tassels and upper leaves, and small ears or heading failure. In China, the prevalence of MRDD has continued to increase with global warming and the reduced use of pesticides, which significantly reduces yield and results in quality deterioration in maize^2,3^. To date, the use of resistant maize varieties remains the most effective and economical way to control this disease. Thus, mapping and cloning genes underlying the Quantitative Trait Loci (QTLs) conferring resistance to MRDD are necessary for the development of resistant hybrids.

In recent years, a few studies focusing on resistant germplasm screening under natural infection conditions have been conducted in China^9–13^. These investigations suggested that there is a lack of germplasm resources that are immune to MRDD, but a limited number of highly resistant lines were identified in different environments, and were mainly derived from a US hybrid, P78599. However, significant differences in resistance among various germplasm resources have been reported^9,10,13,14^.

Previous studies have indicated that the inheritance of MRDD resistance in maize is quantitative in nature^14–16^. Several QTLs have been identified using F_2:3_ segregating populations and recombined inbred lines (RIL) derived from crosses between susceptible and resistant lines. The loci of the identified QTLs from the different resistant lines vary^2,17–23^. However, some QTLs confer similar genetic effects in resistance among different maize inbred lines, such as the major QTL on chromosome 8 (binn8.03) that is associated with a 24.2–39.3% decrease in disease severity and is inherited in a recessive manner^22^.

Although a number of resistant QTLs have been identified, the majority are derived from the US hybrid P78599, including Qi 319 and X178^24,25^. Therefore, the identified QTLs may represent only a small portion of the available genetic resistance to MRDD in maize. Genome-wide association studies (GWAS) allow for the detection of natural variations in complex genetic traits at a relatively high resolution in plants^26–28^. Recent studies have employed GWAS to detect MRDD resistance-related loci in maize using single-nucleotide polymorphism (SNP) markers covering the entire maize genome^25,29,30^. Similar to the findings of linkage analysis, GWAS indicated that each chromosome has a locus/loci associated with MRDD resistance. However, no MRDD resistance-related locus has been successfully cloned from maize to date.

To identify the genes/loci that confer resistance to MRDD, the present study employed a novel germplasm resource that exhibits MRDD resistance. The combined deep sequencing of two DNA pools containing extremely resistant or susceptible F_2_ individuals and traditional linkage analysis using F_2_ populations derived from a cross between a maize susceptible inbred line, S221, and a maize resistant inbred line, K36, were conducted. A genomic region associated with dominantly inherited MRDD resistance was identified on chromosome 6. Our results improve the understanding of MRDD resistance inheritance in maize and should inform the development of breeding schemes for MRDD-resistant maize lines.

## Results

### Evaluation and genetic analysis of MRDD resistance

**Figure 1:**
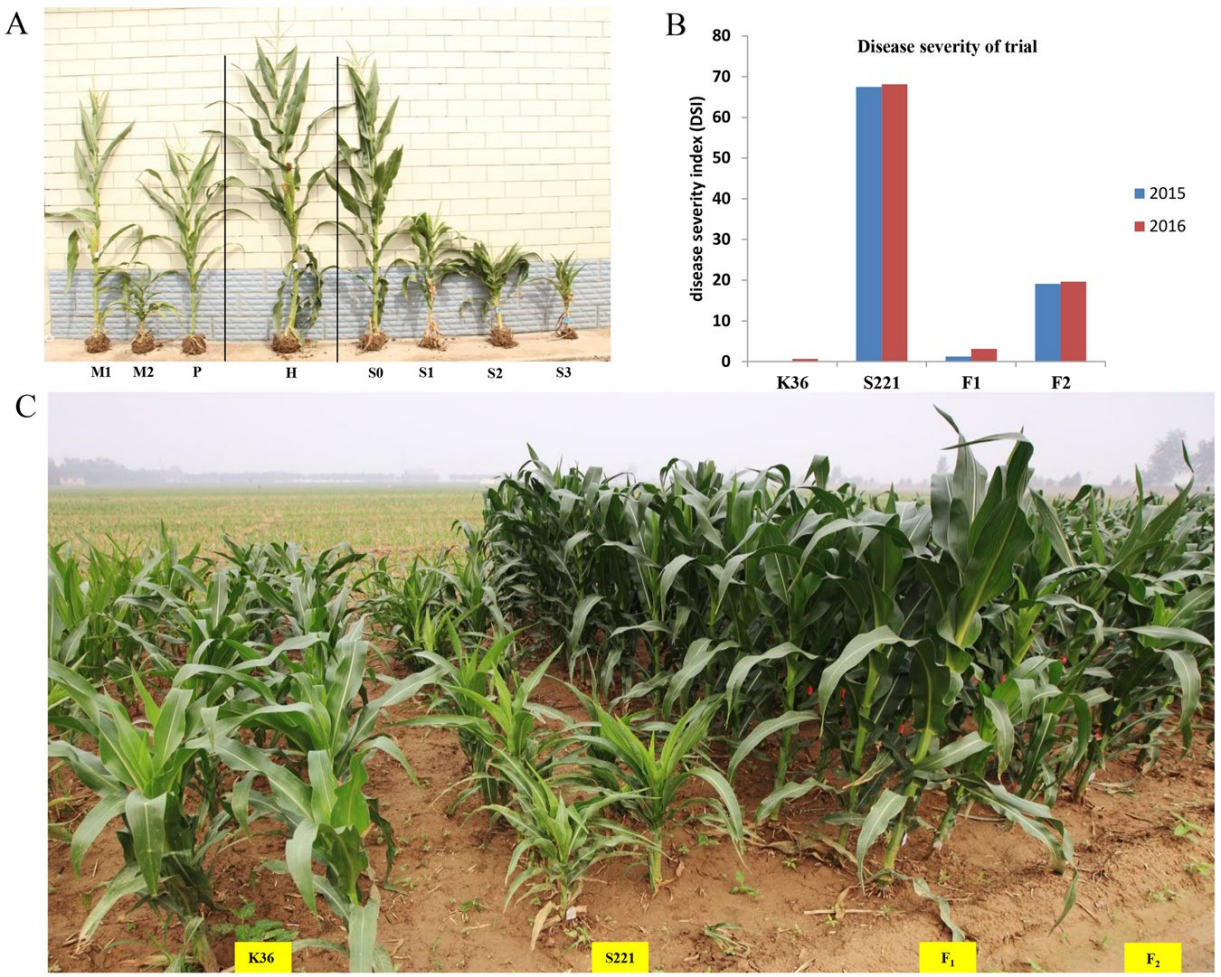
The phenotype of MRDD. (**A)** Parental lines, F_1_ and F_2_ during the powdery period. M1 and M2 represent healthy and MRDD-afflicted S221 plants, respectively; P and H represent K36 plants and hybrid plants, respectively; S0–S3 represent F_2_ plants with MRDD scores of 0–3. (**B**) The disease severity index of parental lines F_1_ and F_2_ in 2015 and 2016. (**C)** Parental lines, F_1_ and F_2_ during the seeding period. The two columns on the left are K36, the next two columns are S221, and the next two columns are F_1_ and F_2_.

The parental lines, S221 and K36, and their F_1_ and F_2_ progenies were evaluated for MRDD resistance in 2015 and 2016 (Fig. 1A,B). Obvious symptoms of MRDD were observed in the field trial under naturally infested conditions in both 2015 and 2016 at Shijiazhuang; a summer maize-growing region in China. The average disease severity index (DSI) scores of K36 and S221 were 0.32 and 67.82 in the two years, respectively (Fig. 1C and Supplementary Table S1). In the two F_2_ populations planted in 2015 and 2016, respectively, there were more plants with disease scores of 0 or 3 than with scores of 1 or 2 (phenotyping section in Method & Supplementary Table S1). The disease score distributions were similar between the two F_2_ populations. The DSI in F_1_ ranged from 1.25 to 3.13, which is similar to that of the paternal line, K36, which is highly resistant to MRDD, thereby suggesting that the MRDD resistance in F_1_ was derived from K36 in a dominant inheritance pattern (Supplementary Table S1). Meanwhile, resistance segregated in the two F_2_ populations with similar disease incidences of 28.3% and 27.8%, and DSIs of 19.1 and 19.6 in both years (Supplementary Table S1).

To characterize the inheritance pattern of MRDD resistance, the disease resistance scores of the F_1_ and F_2_ individuals were statistically analyzed. The F_1_ plants showed resistance to MRDD, while the F_2_ populations exhibited variation in disease resistance scores. When the F_2_ plants were classified into two groups, namely, resistant or susceptible, the chi-square test indicated that the segregation ratio of the resistant and susceptible plants fitted the expected 3:1 segregation ratio (χ^2^ = 2.917, 3.835, respectively), suggesting that MRDD resistance from K36 was controlled by a single dominant locus (Table 1).

**Table 1:** Chi-square test of the segregation ratio of the F_2_ families.

Year	Number of plants			Expected segregation ratio (R:S)	χ^2^
	Total	Resistant (R)	Susceptible (S)		
2015	513	368	145	3:1	2.917
2016	940	679	261	3:1	3.835

χ^2^ < χ^2^_0.05_ = 3.841.

### Virus detection

**Figure 2:**
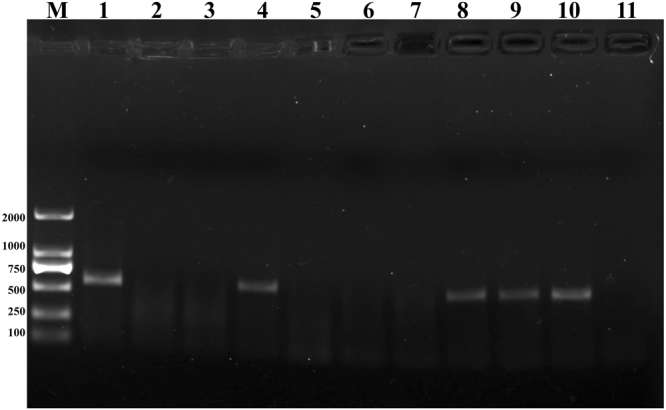
Detection of MRDD in infected and virus-free maize plants. RT-PCR products were visualized after agarose gel electrophoresis with the G-Box gel documentation system (Syngene, Cambridge, UK). M represents a molecular weight marker DL2000 (TaKaRa, Dalian, China) with band sizes indicated on the left of the image (in bps). Lane 1 represents virus cDNA, lane 2 represents MRDD-resistant parent K36, lane 3 represents virus-free MRDD-susceptible parent S221, lane 4 represents MRDD-infected susceptible parent S221-S, lane 5–7 represent F_2_ samples from the R-pool, lanes 8–10 represent F_2_ samples from the S-pool, lane 11 is a no-template control. The MRDD amplicon is 568 bp in size.

MRDD was detected in the maize plants, including the susceptible parent S221-S and the individuals making up the S-pool with dwarf symptoms, but was not detected in the resistant parent, K36, and the 30 plants making up the R-pool (Fig. 2). The real-time (RT)-PCR amplified fragment within the expected size (568 bp) from the cDNA of plants with dwarf symptoms was sequenced. Comparison of the obtained sequences with those deposited in GenBank showed that the sequenced fragment had 94% identity with that of the RBSDV from Wuhan (AJ291706), Jiangsu (KC875238, KM921681), Shandong (JX421771), Anhui (KX660762), China, and 93% identity with the RBSDV from Baoding (DQ407917), China (Supplementary Fig. S1). Additionally, two F_2_ populations from the same cross (S221 × K36) were planted as controls without virus infection. One population consisting of 180 individuals was planted on September 15, 2016 in a protected greenhouse. The other population for the RIL population construction consisting of 846 individuals was sowed on June 20, 2016 to avoid planthopper infection. The plants from both populations showed no dwarf symptoms caused by RBSDV. Together with the high homology between the sequences obtained from samples exhibiting MRDD symptoms and the RBSDV sequences from the maize planting zone of China from GenBank, the results suggested that the dwarf symptoms in the parental varieties and F_2_ individuals were caused by RBSDV infection.

### High-throughput sequencing analysis

**Figure 3:**
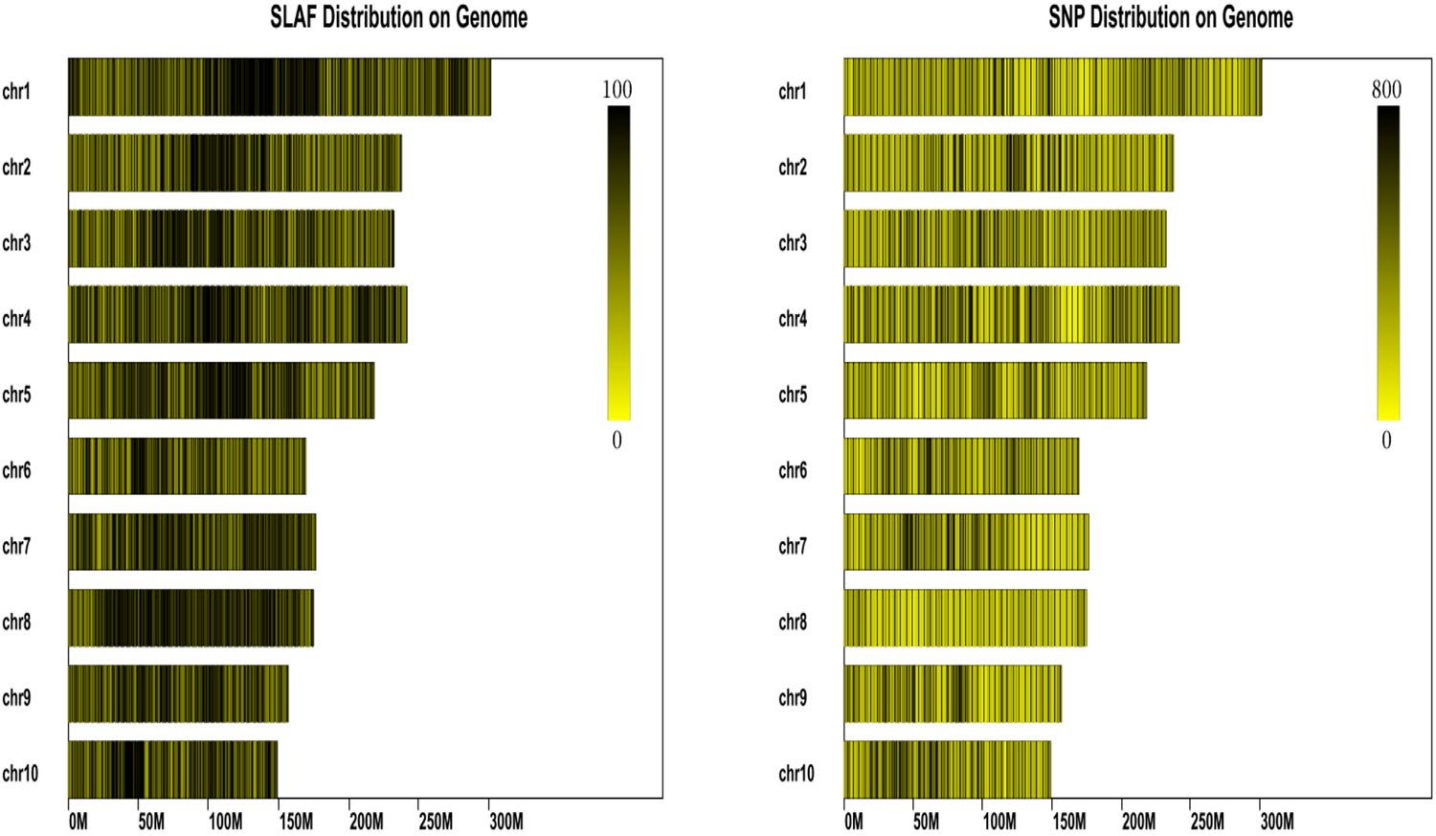
The distribution of SLAFs and SNPs on each maize chromosome. The x-axis indicates the physical position in megabases. The color bar shows the SLAF/SNP density. Dark and light colors denote the SLAF/SNP marker numbers at these loci.

Based on the phenotypic screening for MRDD resistance, four types were classified according to disease severity scores ranging from 0 to 3. From the F_2_ population, two contrasting DNA bulks were constructed by pooling equal volumes of DNA from R plants (disease severity score 0) and S plants (disease severity score 3) to form R and S bulks, respectively. Specific-locus amplified fragment sequencing (SLAF-seq) for the two BSA bulks was conducted, resulting in 12.97 Mb fragments from the two parents and 19.48 Mb fragments from the two bulks after exclusion of the low-quality fragments in the constructed SLAF library (Table 2). When the obtained SLAF-seqs were matched to the reference genome, the results showed that over 92% of the reads could be mapped to the reference genome, and a total of 182,132 high-quality SLAF-tags were developed with an average sequencing depth of 24.89-fold in the parental lines and 32.73-fold in the F_2_ bulks. Approximately 22,644 of the 182,132 SLAF-tags were polymorphic. A total of 709,670 SNPs were detected with ~4% and ~19% heterozygosity in the parental lines and F_2_ bulk pools, respectively (Table 2). Based on the results of the SLAF/SNP marker positioning on the genome, the number of markers on each chromosome were calculated **(**Supplementary Table S2**)**. Of these markers, a total of 181,235 SLAFs and 707,448 SNPs were localized to specific chromosomes. The distribution diagrams for the SLAF/SNP markers on each chromosome were drawn based on their position on the genome (Fig. 3). The markers were distributed equally on each chromosome, and the maize genome was successfully simplified by using this restriction site associated DNA sequencing approach.

**Table 2:** Summary of the developed SLAF-seq coverage and SNP data for each sample.

Sample	Total reads	Total map (%)	SLAF number	polySLAF number	SNP number	HeterLoci number	Heterozygosity ratio (%)
K36	5,586,946	92.47	150,739	21624	394,247	16,232	4.11
S221	7,379,473	92.31	155,642	21807	422,079	18,273	4.32
S-pool	9,160,853	92.50	178,392	22615	535,203	100,032	18.69
R-pool	10,315,083	92.45	178,622	22633	539,605	102,646	19.02

Legend: SLAF number: total number of developed SLAF tags in each sample; SNP number: total number of SLAFs that contain SNPs in each sample; Heter ratio (%): heterozygosity of SNPs in each sample; polySLAF: number of SLAF tags that contain SNPs.

### Association analysis

**Figure 4:**
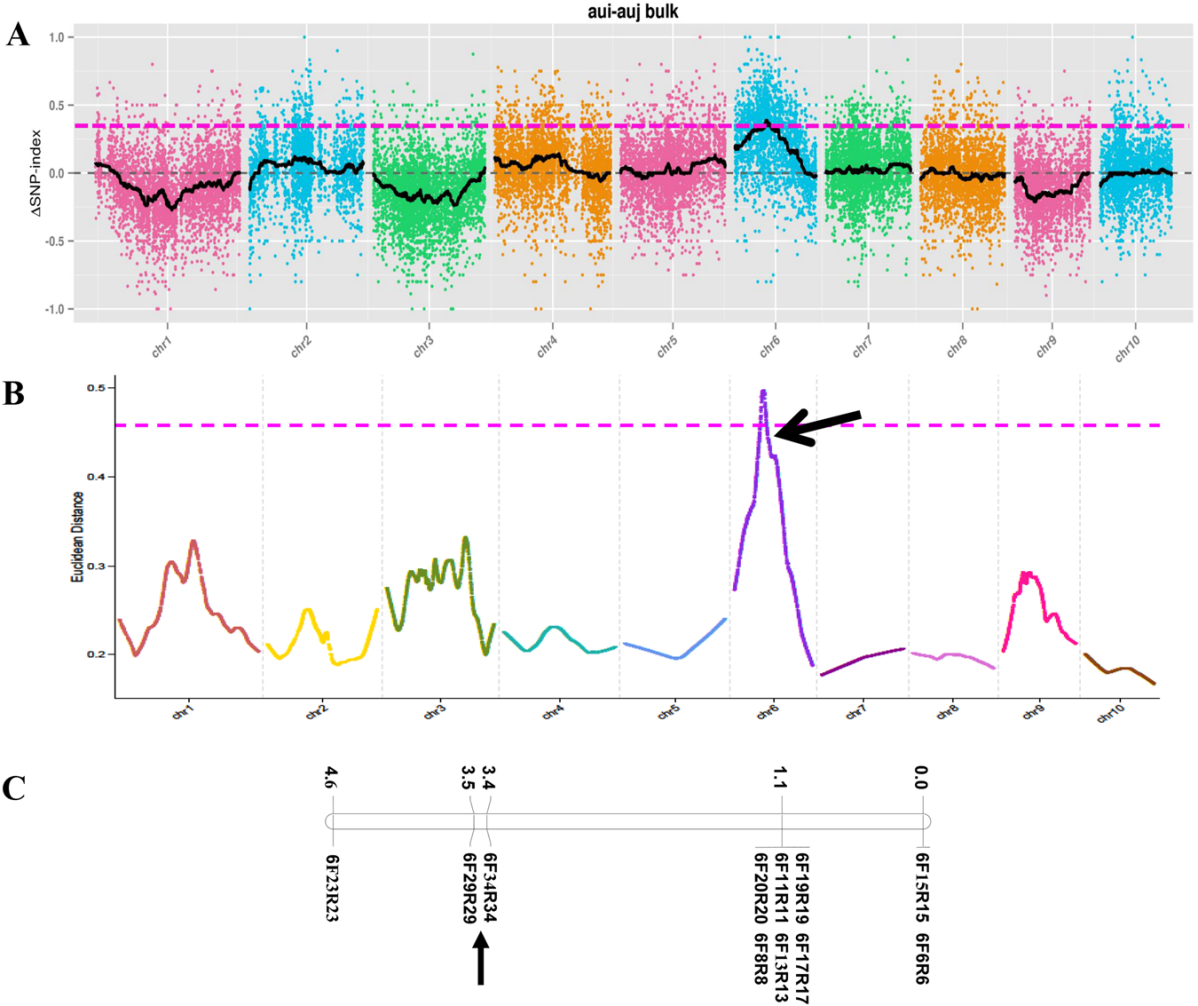
Identification of an MRDD resistance-related region. (**A**) Δ(SNP-index) plot. The x-axis represents the chromosome position, and the y-axis represents the Δ(SNP-index) values. The small colored dots indicate Δ(SNP-index) values. The black lines are the values of the fitted Δ(SNP-index) by association analysis. The pink dotted line is the threshold value (0.3425). (**B**) The Euclidean distance (ED) plot. The x-axis represents the chromosome position, and the y-axis represents the ED values. The pink dotted line is the threshold value (0.458). The putative MRDD resistance-associated genomic region is indicated by an arrow. (**C**) Linkage map of the region harboring *rmrdd6* on chromosome 6. The linkage map was constructed with 11 markers, encompassing a region 18,658,337 bp in size. The genetic distances (cM) between adjacent markers are shown on the left, whereas the names of the mapped markers are on the right.

The segregation ratio of resistance to MRDD in the F_2_ population indicated that the trait is controlled by a dominant locus. Thus, the association threshold of the ΔSNP-index value is expected to be 0.67. By examining the ΔSNP-index plot, peak regions above the threshold value were defined as association regions. After calculations and fitting analysis, the ΔSNP indices of the two bulks were obtained (Fig. 4A). No region exceeded the theoretical threshold on each chromosome, although a region on chromosome 6 was found near the theoretical threshold (Fig. 4B). When the threshold was reduced to 0.34 (quantile 99 of the ΔSNP-index), a possible trait-related candidate region was obtained, which spanned 12.99 Mb (61,960,714 to 74,957,430 bp) on chromosome 6 of the reference genome and harbored 374 genes.

The Euclidean distance (ED) method was employed to further explore the trait-related region. Initially, 158,151 high-quality SNPs were obtained from a total of 709,670 SNPs by filtering out 551,519 SNP sites with read support less than 4 in the two pools. Then, a total of 59,665 different SNPs were identified from the obtained high-quality SNPs. Finally, after the ED value was calculated, the correlation values were obtained using the local linear regression LOESS method (Fig. 4B). A possible trait-related candidate region was obtained when the threshold was 0.458. This associated region spanned 13.80 Mb from 55,724,783 to 69,523,478 bp on chromosome 6 of the reference genomic sequence, where the candidate region obtained using ΔSNP-index method was also mostly covered.

### SSR analysis

To validate the MRDD resistance-associated genomic region and to investigate recombination among the F_2_ individuals, SSR markers in the target region were selected for linkage analysis. First, 35 SSRs distributed in the associated region were used in the assessment of the two parents. Of these, 11 markers showed polymorphism between the two parents. After 126 individual plants from the F_2_ population were genotyped with the 11 SSR markers, a short linkage map was constructed using the JoinMap 4 program. The 11 markers covered a genetic distance of 4.6 cM on chromosome 6. The major MRDD resistance locus on chromosome 6 was mapped between the 6F29R29 and 6F34R34 markers, which were 0.1 cM apart (Fig. 4C). According to the genomic sequence of chromosome 6 in the B73 genome assembly, the physical position of 6F29R29 starts from 68,396,487 bp, whereas 6F34R34 starts from 73,915,399 bp. The two markers covered approximately 5.52 Mb of the candidate region (Fig. 4C).

A summary of the results from both the association analysis of the SLAF-seq and SSR analysis indicated that an MRDD-resistance locus resides on chromosome 6. The candidate locus was designated as Rmrdd6 (resistance to maize rough dwarf disease on chromosome 6), and is located at the intersection of the three obtained candidate regions, with the physical positions starting from 68,396,487 bp to 69,523,478 bp, encompassing approximately 1.13 Mb, and harboring 32 candidate genes.

### Candidate gene identification

To identify candidate genes in the MRDD resistance-associated region, the initial SNPs within the exonic region between the two parents were analyzed and variations were annotated (Supplementary Table S3). A total of 32 genes were included in the associated region. Gene ontology (GO)-based gene functional enrichment analysis was performed, and the 32 genes were characterized according to cellular component, molecular function, and biological process. The results revealed that the most enriched terms were in the category of cellular component, such as integral to cell wall (GO:0005618), anchored component of plasma membrane (GO:0046658), chloroplast envelope (GO:0009941), glycosylphosphatidylinositol (GPI) transamidase complex (GO:0042765), and chloroplast (GO:0009507). Of the 32 genes, 9 possess known or hypothetical functions, and 23 genes encode uncharacterized proteins based on GO analysis and the publicly available B73 annotated genome (http://plants.ensembl.org/Zea_mays/Info/Index). Among the 9 annotated genes, GRMZM2G161673 is predicted to encode magnesium protoporphyrin IX methyltransferase and has methyltransferase activity in chloroplasts. GRMZM2G471321, also known as the *Tangled1* gene, is required for the spatial control of cytoskeletal arrays that are associated with cell division during maize leaf development^31^. GRMZM2G156422 encodes a transmembrane protein that is essential for retrograde transport in the trans-Golgi network. The NAC-like gene GRMZM2G091490 is predicted to encode a transcription factor that plays an important role in the regulation of plant growth and development, hormone regulation, and responses to various stresses^32,33^. Another NAC-like gene GRMZM2G110983 is predicted to encode a protein containing a ubiquitin-conjugating enzyme E2 catalytic (UBCc) domain that is involved in a ubiquitin-mediated protein degradation pathway^34^. GRMZM2G478892 encodes a protein belonging to the O-glycosyl hydrolase family 17 and hydrolyzes O-glycosyl compounds in the cell wall matrix of plants, and also has diverse roles in plant defense and plant development^35^. GRMZM2G175867, a member of the DEAD-box ATP-dependent RNA helicase family, plays important roles in RNA metabolism and abiotic stress responses in chloroplasts and mitochondria^36^. GRMZM2G178602 encodes a glycosylphosphatidylinositol (GPI) transamidase component Gpi16 subunit family protein that is involved in anchor biosynthesis, acting as an anchor for the attachment of cell surface proteins to the cell membrane^37^. GRMZM2G178616 encodes phosducin-like protein 3 that plays a central role in cell division in the microtubule assembly pathway^38^.

Furthermore, 7 SNPs with nonsynonymous mutations were identified in this genomic region, corresponding to 5 genes that might be related to MRDD resistance. In particular, GRMZM2G384564 is more interesting because it had 3 SNPs with nonsynonymous mutations in the paternal line K36.

## Discussion

### Accurate phenotypic evaluation

Accurate phenotypic evaluation is critical for marker-trait association analyses^39^. As with all viral diseases, MRDD requires interactions among an effective vector, the virulent pathogen, a susceptible host, and an appropriate environment^40^. Wheat is one of the preferred hosts for the planthopper insect vector (*L*. *striatellus*) because it provides an optimum environment for the insects to complete their development, after which they then migrate to adjacent maize crops. In the present study, natural infection was used because the virus causing MRDD is transmitted in a persistent propagative manner, and the prevalence of MRDD in the experiment was attributable to the extensive sowing of wheat prior to that of maize. Virus detection showed that the dwarf symptoms in the infected plants were caused by MRDV. Therefore, natural infection ensures accurate phenotypic evaluation. In 2015 and 2016, 98% of the plants of maternal line S221 were infected, which is indicative of relatively stable and uniform infection conditions and implies that the results of the MRDD resistance evaluation were appropriate for further marker-trait association analysis. Our study further proved that natural infection is a suitable method for evaluating MRDD resistance^17,22,23^.

### Genetic analysis of MRDD resistance

In the present study, the paternal line K36 displayed consistent resistance, whereas the maternal line S221 showed susceptibility. Although there are several infected plants in their F_1_ populations, the majority, like K36, exhibited MRDD resistance. The F_2_ populations in the genetic analysis, consisting of an appropriate number of individuals, ensured that the results of the chi-square test were accurate. The segregation ratio of resistant and susceptible plants of the F_2_ population was 3:1, indicating that a single dominant gene controlled the resistance trait from K36. Our results differ from those of previous reports whereby MRDD resistance in maize was found to be controlled by several genes, each with a small effect^14–16^. Different resistant maize germplasm resources display variable resistance to MRDD^9–13^. Several characterized highly resistant inbred lines, including Qi319 and X178, derived their resistance from the US hybrid P78599, and consequently, several linkage-based mapping studies in China identified QTLs that control resistance^2,21,22^. The identification of a dominant gene in K36 in this study broadens the genetic basis and enriches our understanding of the genetic mechanism underlying differences in MRDD resistance.

### Effectiveness of the SLAF-seq analysis and identification of resistance-associated regions

Traditional gene mapping and map-based cloning methods have facilitated the identification of molecular markers that flank and co-segregate with a specific locus^41^. BSA is an efficient method for the rapid identification of molecular markers linked to the target gene or genomic region in two bulks showing clear phenotypic differentiation^42^. However, the availability of DNA markers is the main factor limiting BSA effectiveness^43^. Next-generation sequencing technologies provide a comprehensive and cost-effective means of unraveling the genetic diversity of genomes for accelerating gene mapping and isolation. The combination of SLAF-seq and BSA circumvents the limitation of DNA marker availability and does not require complete genotyping. This strategy has been successfully applied to various plant species including wheat^44^, rice^43,45^, sorghum^46^, and sunflower^47^. In the present study, both BSA-SLAF-seq and linkage analysis were conducted to detect resistance-related genomic regions. More than 182,132 SLAFs were developed and 709,670 SNP markers were identified, all of which were of high quantity and quality. The SLAFs and SNPs were distributed across each chromosome. The integrity and accuracy of these markers are relatively high compared to the 54,788 and 56,635 SLAFs developed by Yu and Xia when they identified genes associated with defective, pale green bundle sheaths and inflorescence meristems in maize, respectively^48,49^. Therefore, the obtained markers provided sufficient data for the identification of candidate MRDD resistance-associated regions in this study.

SLAF-seq analysis identified one resistance-associated genomic region on chromosome 6 encompassing 61,960,714 bp to 69,523,478 bp. Subsequently, SSR markers were selected for linkage analysis to validate the identified region. The linkage results further confirmed the associated genomic region on chromosome 6 and narrowed the region down to 1.13 Mb, encompassing the genomic region from 68,396,487 bp to 69,523,478 bp. Previous studies have identified numerous QTLs conferring resistance to MRDD through linkage mapping or genome-wide association analysis. Additionally, a few of these identified QTLs were consistently associated with MRDD in various studies, such as a major QTL locus on chromosome bin 8.03^2,18,22,23^. Although some QTLs were also identified on chromosome 6^17,25,30,50,51^, these did not overlap with the genomic region identified in the present study. Therefore, our findings provide new insights into the genetic architecture of MRDD resistance in maize. The identification of markers that are significantly associated with traits of interest is mainly used for MAS in plant breeding programs. In the present study, the Rmrdd6 that confers resistance to MRDD was mapped to a 1.13-Mb interval. Two SSR markers, 6F29R29 and 6F34R34, were tightly linked to the MRDD resistance gene, which can be efficiently used in the MAS of MRDD resistance and accelerate the improvement of maize breeding.

### Potential candidate genes resistant to MRDD

The ultimate goal of gene mapping is to obtain the target candidate gene. In this study, the GRMZM2G384564 gene was of great interest to MRDD resistance, as three non-synonymous mutations were identified in the paternal line K36 when compared to reference B73 and maternal line S221. Whether these mutations conferred resistance to MRDD in K36 requires further validation. GRMZM2G384564 is a maize-specific gene, but its function remains unclear. Therefore, further assessment of this gene is warranted. Dwarfing and dark green leaves with white enations are the major symptoms of MRDD. Previous studies on the response of maize to RBSDV infection have revealed that the expression patterns of cell wall- and development-related genes, chloroplast-related genes, and disease resistance- and stress-related genes are dramatically altered^52,53^. Some genes were identified within the genomic region associated with MRDD resistance, such as cell wall-related genes GRMZM2G478892 and GRMZM2G471321, and chloroplast-related genes GRMZM2G178602, GRMZM2G156422, GRMZM2G175867, and GRMZM2G161673.

In addition to the cellular component genes, the ubiquitin-related genes and genes involved in the ubiquitin biosynthesis pathway were influenced in maize infected with MRSDV^52,53^. Previous studies have also shown that protein ubiquitin-mediated degradation is involved in plant disease resistance^54,55^. In the MRDD resistance-associated genomic region, two ubiquitin-related genes, namely GRMZM2G091490 and GRMZM2G110983, were predicted to be associated with the regulation of plant growth and development and plant defense^32,33^. Confirmation of the roles and functions of these candidate genes is necessary to facilitate a better understanding of how these genes contribute to MRDD resistance in maize and, more generally, improve our knowledge of the genetic mechanisms underlying resistance to viruses in higher plants. Additional studies elucidating the mechanism of interaction between maize MRDD resistance candidate genes and RBSDV infection are necessary.

Taken together, the results of the present study have provided novel insights into the genetic architecture of MRDD resistance, which is controlled by a dominant gene in maize line K36. The susceptibility to MRDD may thus easily be controlled by identifying a resistance gene in the host that renders resistance to the disease. Furthermore, two molecular markers, 6F29R29 and 6F34R34 on chromosome 6, were determined to be tightly linked to the resistance gene. These two markers, together with the highly resistant inbred line K36, may potentially be utilized in MRDD resistance breeding programs.

## Materials and Methods

### Plant materials

The parents used in this study are elite inbred lines with high general combining ability. The maternal line S221, which is highly susceptible to MRDD, was derived from the Reid group. The paternal line K36, which is highly resistant to MRDD, was selected from naturally mixed pollination of the US hybrid P78646 and Y7865. The F_1_, F_2_ progenies, which were selfed, were obtained by crossing S221 with K36. Together with the two parents, these comprised the dataset employed in this study.

### Field evaluation

The MRDD resistance of all the parental plants and their progenies were evaluated under natural infection conditions in 2015 and 2016 in Shijiazhuang, where MRDD is prevalent. The previous crop in the experimental field was wheat, which constitutes a reservoir for both vector and virus. Forty plants of each parent and 40 F_1_ individuals were planted in 2-row plots in each year. The F_2_ populations included 513 individual plants in 2015 and 940 in 2016. All plants for resistance identification were sowed on May 11 of each year during the transmission period of the causal virus by its insect vector, *L*. *striatellus*. The other two F_2_ populations from the same cross and their parental inbred lines were planted as controls without virus infection. One population consisting of 180 individuals was planted on September 15, 2016 in a protected greenhouse. The other population for RIL population construction consisting of 846 individuals was sowed on June 20, 2016 to avoid planthopper infection. All seeds were sowed by hand in rows of 0.6 m width and 5 m length. The seeds were coated with chemical agents pre-sowing to comprehensively control underground pests and pathogenic bacteria during the germination and seedling stage. Chemical agents included bactericides of fludioxonil, metalaxyl-M, and difenoconazole, and pesticides of Phoxim and Chlorpyrifos. The experimental field comprised cinnamon soil. Within the top-20-cm of the soil layer, the soil organic matter content was 16.2–16.9 g/kg, total nitrogen 1.14–1.20 g/kg, total phosphorus 2.1–2.5 g/kg, alkaline hydrolysis nitrogen 128–144 mg/kg, effective phosphorus 24.40~27.84 mg/kg, and available potassium 163.5~177.4 mg/kg. The amount of base fertilizer (composed N-P_2_O_5_-K_2_O (N/P/K = 22/8/10) used was 60 g/m^2^ without top dressing throughout the plant growing season. Irrigation and weeding during growth were the same as in the conventional field.

### Phenotyping and chi-square test

The disease severity of all tested maize plants was assessed during the maturity stage and used a four-point disease severity scheme: 0, no symptoms; 1, mild symptoms such as presence of galls on the abaxial side leaves, or enations; 2, shortened superior internodes, enations, and ‘hockey pole’ ears; and 3, severe dwarfing, enations, and small ears with few or no kernels. Then, the disease incidence and DSI of each plot were calculated using the following equation: DSI (%) = ∑(rating scale score × number of plants with that rating scale) × 100/3 × total number of plants as described by Di Renzo^16^. The F_2_ population was subjected to a chi-square goodness-of-fit test.

### Virus detection by RT-PCR

A total of 63 plant samples including the parental materials (K36, S221-S with MRDD symptoms, and S221 planted in a greenhouse without planthopper infection) and a subset of 60 F_2_ individuals making up the R- and S-pool for deep sequencing were analyzed for the presence of the virus by RT-PCR^4,56^, with modifications. Approximately 100 mg of maize leaves were ground in liquid nitrogen and mixed with 1 mL of TRIzol reagent (Invitrogen, CA, USA) for RNA extraction. The concentration and quality of RNA were estimated using a NanoDrop spectrophotometer (Thermo Scientific, Waltham, MA, USA) and gel electrophoresis. Approximately 2 μg of total RNA was used for cDNA synthesis using a Superscript III first-strand synthesis system (Invitrogen, CA, USA) and random primers (New England Biolabs, UK) following the manufacturer’s instructions. The virus cDNA was kindly provided by Dr. Jianfeng Wei of the Institute of Crop Science, Chinese Academy of Agricultural Sciences. A set of published primers (up-5′AGCGGAGAACGTTTGGATC3′/dn-5′TTAACAACAGCAGCTTCACC3′) designed from the highly conserved regions (568 bp) within the viral genome was used for virus detection^4,56^. RT-PCR was performed in 96 well-plates; each reaction was conducted in a 20-μL reaction containing 7.5 nM primers and 2.5 μL of cDNA. The CFX96 Connect™ Real-Time PCR Detection System (Bio-Rad, USA) was used with the following conditions: an initial denaturation at 95 °C for 5 min, followed by 40 cycles of 94 °C for 30 s, 55 °C for 30 s, and an extension at 72 °C for 30 s. The *Zm-Actin* gene was used as an internal control. The primer sequences of the *Actin* gene were up-5′CACCTTCTACAACGAGCTCC3′/dn-5′CAGTCAGGATCTTCATGAGG3′ ^57^. The PCR products were assessed for purity and size by ethidium bromide staining after agarose gel electrophoresis (1.5% agarose, TAE buffer) and sequenced to confirm the presence of the target DNA fragment.

### DNA extraction and pool construction

Based on disease severity, 30 extremely resistant (disease score 0) individual plants and 30 extremely susceptible (disease score 3) individual plants from the F_2_ population were selected and grouped as two bulks, namely resistant pool (R-pool) and susceptible pool (S-pool), for the BSA of the year 2015^42^. Total genomic DNA was isolated from the young leaves of the two parents and each plant from the two bulks according to Doyle *et al*. (1990), with minor modifications^58^. The DNA quantity and quality were measured with a spectrophotometer (NanoDrop 2000, Thermo Scientific, Waltham, MA USA) and by 1.5% agarose gel electrophoresis. The bulked DNA samples were prepared by mixing an equal ratio of DNA from each plant at a final concentration of 30 ng/μL. These DNA samples were used for SLAF-seq analysis.

### SLAF library construction and sequencing

The genome sequence of *Z*. *mays* (2,500 Mb) was used as a reference sequence (ftp://ftp.ensemblgenomes.org/pub/plants/release-24/fasta/zea_mays/). To obtain an even distribution of SLAFs, an initial simulated restriction enzyme digestion was conducted with the maize genomic reference sequence to optimize conditions for SLAF yield. Based on the results of the simulated restriction enzyme digestion, the genomic DNA of the two parents and pools were digested using the appropriate restriction enzyme combination of *HaeIII* and *Hpy166II*, with the genomic DNA of rice (*Oryza sativa*) used as a control, to assess the effectiveness of the enzyme digestion. All subsequent SLAF-seq procedures were performed according to Sun *et al*.^59^ with minor modifications. DNA fragments 414–444 bp in length were selected as SLAFs and prepared for pair-end sequencing on an Illumina High-seq. 2500 sequencing platform (Illumina, Inc.; San Diego, CA, USA) at the Beijing Biomarker Technologies Corporation (http://www.biomarker.com.cn).

### Sequencing data analysis and SLAF definition

To ensure the quality and effectivity of the original sequencing data, the 100-bp flanking regions of the raw reads of 414–444 bp in length were filtered out and used for data evaluation. SOAP2 (Short Oligo Nucleotide Alignment Program 2) was employed to map these onto the reference genome after correction (ftp://ftp.ensemblgenomes.org/pub/plants/release-24/fasta/zea_mays/)^60^. The SLAF groups were generated according to the reads that were mapped to the same position. Alleles were assigned to each SLAF based on the results of the Burrows-Wheeler Aligner (BWA) evaluation. SLAF-tags were defined as parent sequences, and bulks were genotyped by similarity to the reference genome sequence. The GATK (Genome Analysis Toolkit) software (https://software.broadinstitute.org/gatk/best-practices?bpm=DNAseq#variant-discovery-ovw) was used for SNP detection. First, GATK was used in conducting local realignments according to the localization results of the sequencing reads onto the reference genome, and then GATK and Samtools (Sequence Alignment/Map tools) were used for variant SNP discovery analysis. Finally, a set of SNP sites was obtained by selecting the intersection of the SNP discovered by GATK and Samtools to ensure accurate SNP detection.

### Association analysis

The SNP-index is a recently developed association analysis method for finding significant differences in genotype frequencies between pools indicated by Δ(SNP-index)^61^. In the association analysis, auj stands for the R-pool and aui for the S-pool. The Δ(SNP-index) was calculated as follows: SNP-index (auj) = ρx/(ρX + ρx), SNP-index (aui) = ρx/(ρX + ρx), Δ(SNP-index) = SNP-index(auj) - SNP-index(aui), in which ρX and ρx represent the reads of the genotype for a single marker (SNP) in the R-pool and S-pool, respectively. The Δ(SNP-index) was used for the association analysis. To ensure the reliability of the related association regions, ED was also used to identify significantly different markers and evaluate the associated regions according to the method described by Hill^62^. Regions above the threshold were considered as trait-related candidate regions.

### Narrowing down the MRDD resistance-associated regions by SSR assays

Based on the physical locations of the SSR markers developed by Xu *et al*.^63^, 35 SSR markers were selected from the associated region on chromosome 6 (Supplementary Table S4). These primers were synthesized by Sangon Biotech Co. Ltd., Shanghai, China. To obtain polymorphic SSR markers between S221 and K36, the selected markers were first surveyed using the two parental lines. The identified informative SSR markers were then used in genotyping the F_2_ individuals. PCR was performed in 96-well plates using 20-μL reactions containing 100 ng of DNA template, 1 pmol of each primer, and 2× Tag PCR StarMix with loading buffer (Invitrogen Biotechnology Co. Ltd., Shanghai, China). PCR conditions were as follows: 1 cycle at 95 °C for 10 min, followed by 36 cycles at 95 °C for 30 s, annealing temperature at 53–58 °C for 30 s, and elongation at 72 °C for 30 s, then 1 cycle consisting of a final elongation step at 72 °C for 10 min. All PCR products were evaluated by denaturing polyacrylamide gel electrophoresis (6% polyacrylamide) using DYY-12C (Liu-Yi, Beijing, China) followed by silver staining. The genotype of each SSR marker was surveyed. Then, the obtained genotype data were analyzed with MAPMAKER/EXP version 3.0b^64^, using the Kosambi map function to calculate genetic distances. Linkage was determined at a logarithm of the odds (LOD) threshold of 3.0, with a maximum map distance of 50 centiMorgan (cM).

### GO analysis of selected candidate genes

To annotate the genes in the MRDD resistance-associated region, BLAST was used to compare these with genes in the NCBI non-redundant (NR), Swiss-Prot, GO, Clusters of Orthologous Groups of proteins (COG), and Kyoto Encyclopedia of Genes and Genomes (KEGG) databases, respectively. All genes that fell into three categories, including biological process, cellular component, and molecular function, were filtered out based on the statistical information described by Harris *et al*.^65^. The candidate genes were selected after the analysis of the relationship between the functions of the genes and resistance to MRDD.

## Electronic supplementary material

Supplementary information
