# Supplementary material for: Identification of a locus conferring dominant resistance to maize rough dwarf disease in maize
Source: Sci Rep. 2018 Feb 19;8:3248. doi: 10.1038/s41598-018-21677-3 (PMC5818611; doi:10.1038/s41598-018-21677-3)
Supplement: Supplementary file 1 — Supplementary information [file 41598_2018_21677_MOESM1_ESM.pdf]

Identification of a locus conferring dominant resistance to maize rough dwarf disease in maize

Ronggai Li*, Wei Song, Baoqiang Wang, Jianghao Wang, Dongmin Zhang, Quanguo Zhang, Xinghua Li, Jianfen Wei, Zengyu Gao

Supplementary Information

1. **Supplementary Figure S1** Multiple sequence alignment showing identity between S221-S and RBSDV isolated from different maize planting locations in China. S221-S: parental plant showing MRDD symptoms in the present study. The GenBank accession numbers AJ291706, KC875238 and KM921681, JX421771, KX660762, DQ407917 are rice black streaked dwarf virus isolated from Wuhan, Jiangsu, Shandong, Anhui, Baoding China, respectively.

```
S221-S      1    GATCATATAAAATAGAAGAATTGACAATTAAAAACGACCAACCAAATAGAAACACAAATTCATCGACTTCTCAATCTAATGAAAATCGTC  90
AJ291706    3    ..............................................................................C...........  92
MRDV_cDNA   3    ..............................................................................C...........  92
KC875238    3    ..............................................................................C...........  92
JX421771    3    ..............................................................................C...........  92
KX660762    3    ..............................................................................C...........  92
KM921681    3    ................................................................A.............C...........  92

S221-S      91   TTTCAACCAAGAAAATCCCCCTACTCGATGACGGCATCTTCGAACTTCTGAACTACCTTATTGATGGAACCAACTTCGACAAAACTTGCT  180
AJ291706    93   ..........................................................................................  182
MRDV_cDNA   93   ..........................................................................................  182
KC875238    93   ........................................T.................................................  182
JX421771    93   ..........................................................................................  182
KX660762    93   ..........................................................................................  182
KM921681    93   ..........................................................................................  182
DQ407917    1                                        ......................................................  54

S221-S      181  ATTGTGGTTTTAATTATTCTCATCTCCCTAACCTTGAACGTGACTTTAACGTTGCATCTGTTTATGTCCGCGAGAATTTTG-GTCTATAA  269
AJ291706    183  ...........................................................A.....................-AAT...-G  270
MRDV_cDNA   183  ...........................................................A.....................A..-...-G  270
KC875238    183  ...........................................................A.....................A..-...-G  270
JX421771    183  ...........................................................A.....................A..-...-G  270
KX660762    183  ...........................................................A.....................-AAT...-G  270
KM921681    183  ...........................................................A.....................A..-...-G  270
DQ407917    55   ...........................................................A.......T.............A..-...-G  142

S221-S      270  CATTTCTG-GAA---AGATTTGAAAGATTTGGATAGAAAGGCTTGTTTATTGTTGAAATCTGCTGATTTTACTTTGCTTCTAGAATATAC  355
AJ291706    271  ..---...AA..CTT..............AT......C....AAA.A..AGCG........C............................  357
MRDV_cDNA   271  ..---...AA..CCT..............AT....A.C....AAA.A..AGCG........C............................  357
KC875238    271  ..---...AA..CCT..............AT......C....AAA.A..AGCG........C............................  357
JX421771    271  ..---...AA..CTT..............AT......C....AAA.A..AGCG........C............................  357
KX660762    271  ..---...AA..CTT..............AT......C....AAA.A..AGCG........C............................  357
KM921681    271  ..---...AA..CTT..............AT......C....AAA.A..AGCG........C............................  357
DQ407917    143  ..---...AA..CTT..............AT......C....AAA.A..AGCG........C............................  229

S221-S      356  TCTTAAACCATCATCAGAAACCGATCCTGCTATTCCAGAGAATGAGAAAGAAGAGAATGCTAAACCAGTTACTCCTAAAGTTGTCACTCC  445
AJ291706    358  ..........................................................................................  447
MRDV_cDNA   358  ..........................................................................................  447
KC875238    358  ...........................................................T..............................  447
JX421771    358  ............................A.............................................................  447
KX660762    358  ............................A.............................................................  447
KM921681    358  ...................G........A.............................................................  447
DQ407917    230  ............................A.............................................................  319

S221-S      446  AAAAGAAGAGAAGAAAACAGTTGAAATGTCATTGTTGCCAATTCTGAACCGTGAATCTGAAGAATCTTTAAGCTCTGAAATTCTTGAAGG  535
AJ291706    448  ..........................................................................................  537
MRDV_cDNA   448  ..............................G...........................................................  537
KC875238    448  ..........................................................................................  537
JX421771    448  ..............................G...........................................................  537
KX660762    448  ..............................G...........................................................  537
KM921681    448  ..............................G...........................................................  537
DQ407917    320  ..........................................................................................  409

S221-S      536  TGAAGCTGC   544   Identity (%)
AJ291706    538  .....       542        94
MRDV_cDNA   538  .....       542        94
KC875238    538  .....       542        94
JX421771    538  .....       542        94
KX660762    538  .....       542        94
KM921681    538  .....       542        94
DQ407917    410  .........   418        93
```

2. **Supplementary Table S1** MRDD disease severity in the parent lines (K36 and S221) and F_1 and F_2 progenies

	Number of plants in 2015				Number of plants in 2016			
Disease score	K36	S221	F_1	F_2	K36	S221	F_1	F_2
0	40	1	39	368	39	1	37	679
1	0	0	0	56	1	0	1	90
2	0	9	1	29	0	8	0	50
3	0	30	0	60	0	31	2	121
Total number of diseased plants	0	39	1	145	1	39	3	261
Total number of plants	40	40	40	513	40	40	40	940
Disease incidence (%)	0	97.5	2.5	28.27	2.5	97.5	7.5	27.77
Disease severity index (DSI)	0	67.5	1.25	19.10	0.63	68.13	3.13	19.61

3. **Supplementary Table S2** The distribution of markers on each chromosome

Chromosome	Number of SLAFs	Number of SNPs
Chromosome 1	27,423	97,890
Chromosome 2	19,484	83,762
Chromosome 3	19,592	86,975
Chromosome 4	21,635	92,253
Chromosome 5	19,050	69,569
Chromosome 6	13,342	61,784
Chromosome 7	16,145	60,557
Chromosome 8	17,324	40,477
Chromosome 9	13,850	53,106
Chromosome 10	13,390	61,075
Other	897	2,222
Total	182,132	709,670

4. **Supplementary Table S3** Summary of different SNPs in the candidate region between parents

Chromosome	Position (bp)	Ref (B73)	S221	K36	Codon change	Amino acid[a] change	Gene ID
6	62,078,047	G	T	G	tGg/tTg	W/L	GRMZM2G444383
6	62,112,558	C	C	A	Ctt/Att	L/I	AC186583.3_FG004
6	64,074,905	A	A	G	aTt/aCt	I/T	GRMZM2G124972
6	67,811,315	G	C	G	Gtg/Ctg	V/L	GRMZM2G700955
6	69,248,287	A	A	G	Atg/Gtg	M/V	GRMZM2G384564
6	69,248,324	C	C	T	cCa/cTa	P/L	GRMZM2G384564
6	69,248,432	T	T	C	cTc/cCc	L/P	GRMZM2G384564

AA: amino acid

5. **Supplementary Table S4** Information on the SSR markers used in linkage analysis in this study

Marker name	Position on chromosome 6 (bp)	Forward primer (5'-3')	Reverse primer (5'-3')
6F4R4	55,822,325-55,822,566	CACTCAGGGTCTGCTTGGAG	CTCTCTCTATCCTAAAATAGTAGTCGT
6F5R5	55,950,898-55,951,240	CAAAGGGTCGTTTGGGCCA	TTCAACCCGAGGTTTGGAGC
6F6R6	56,058,408-56,058,549	TCTATGACACGTGTGGGCAC	TGTGTTTGTATGACTCCCCCT
6F7R7	56,335,833-56,335,956	ACTACACGACTCCGACTCGA	TTGAACCAGTTTGCAGGGGT
6F8R8	56,452,804-56,452,977	CTACACAGCTTCAAGGCCCA	CCGCTGTTGTTTCTGGCTTC
6F9R9	57,019,210-57,019,447	TGATGCTGCTGCTGCTTTTG	AGCCGAAAAATTCATCACCGA
6F10R10	57,130,660-57,130,868	TGGCCTAGTTTAGAAAAAGAGCT	TCGGTGCAAAGTGGTTTTCC
6F11R11	57,647,144-57,647,245	TCATGGCCGCTTGTGTGTAT	CATCACAAGCAGCACGTACG
6F12R12	57,772,604-57,772,757	TATGTGTGTGCCCTCCTTGC	TTGGCCCACTTTGTTTTGGC
6F13R13	58,683,686-58,683,872	TCCTCCCAATGCAAGGTGTC	CGTAGGGTTGGTTCCGAACA
6F14R14	59,920,064-59,920,534	CTATTCAACCGCCCCCTGTT	ACCGGATCGTTCTCTATGCG
6F15R15	60,113,527-60,113,742	TGCTTGATTGAAGTGCTTGCT	ATGGGCAGTGATGACTCCAT
6F16R16	60,416,993-60,417,205	GTGGGTGTTGCTCAGCATTG	GCAGCGGTAACAACTGAAGC
6F17R17	61,191,473-61,191,687	GGCAGTGCCTGATAGCAAGA	ACCAGTCAACTGCTACGAGC
6F18R18	61,533,190-61,533,389	GAAGCTTGGAGGTAGCGACA	AGCTGTATTACGCGTCGACT
6F19R19	62,656,610-62,656,791	TGCTTTCGTCTCCAGCTAGG	GGCTTCTGACTTTCGGACGA
6F20R20	62,777,760-62,777,922	AGTCAGCAAAGGCCACAGAA	CGAACTGGTGTAACGTGTGC
6F21R21	63,654,542-63,654,669	TCGTTATGAGGGTGATACCAGC	CGTCGTAGCTCGCGAAAATG
6F22R22	64,384,982-64,385,168	ACACAGAGCAACAACATACTCA	TGTCCTTGGATGGTCATGCC
6F23R23	65,076,565-65,076,772	CCGCTGCTTGTGAATCCATG	AACATAACGTAGGTCCGCCC
6F24R24	65,407,726-65,407,919	CTTTACGCTGAAATCCGCCG	GCACCAGATCGCTTGATTGC
6F25R25	65,526,472-65,526,648	CATAGGGGATGAGTTGGGCC	TGCCATGCCGATGTTTTTGT
6F26R26	66,095,500-66,095,709	GGGGTGGTGATCACTCTCAC	AAAATGGAGACGACGGCAGT
6F27R27	66,591,888-66,592,187	TGCTGGAGTTTGTTTGTGCA	GCCCAAGCGGTGTTTTCTTA
6F28R28	67,437,844-67,437,998	CACCGGGGTGCTTGTAGTAG	CACACACACACGTTGACGAC
6F29R29	68,396,487-68,396,671	CACGGACTTAAGGAGCCATGT	CACTCTGAGCTGTGCACTGT
6F30R30	69,764,319-69,764,546	GGTGCATTTTAGTAGCGCCG	TGGGACTAAAAGGCTACCGA
6F31R31	70,996,239-70,996,423	ACTGTGAGGTGGTCAGGACT	AAGCGTTGCTTCTCAGAGGT
6F32R32	72,227,569-72,227,886	AACTTCAACAACGCTTGGGC	CATGGACCCGATCTAGTGCC
6F33R33	72,887,108-72,887,316	TCCGACCATCGTTACATTGGT	ACGTTGAAAAGAACACGCGG
6F34R34	73,915,399-73,915,578	GCCGTGGCTGTAAGAAAAGC	TATGCCGCGAAGTAAAGGCA
6F35R35	74,480,444-74,480,662	TTCATCAACCGCACATACGT	CGCACGTCTCCTTTTCACTTC
